# Anisodamine hydrobromide reduces 28-day mortality in septic shock: a randomized controlled

**DOI:** 10.3389/fphar.2026.1852584

**Published:** 2026-09-22

**Authors:** Xueting Yang, Hongqiong Peng, Tianmeng Pan, Ting Xie, Lei Deng, Ping Zhou, Yijin Chen, Mingxin Xie, Han Zeng, Xia Zeng, Ting Wang

**Affiliations:** 1 Emergency Intensive Care Unit, Sichuan Provincial People’s Hospital, University of Electronic Science and Technology of China, Chengdu, Sichuan, China; 2 Department of Critical Care Medicine, The First People’s Hospital of YiBin, Yibin, Sichuan, China; 3 Endocrinology Department, Emergency General Hospital, Beijing, China

**Keywords:** anisodamine hydrobromide, lactate clearance, mediation analysis, randomized controlled trial, sepsis, septic shock

## Abstract

**Background:**

This randomized controlled trial evaluated whether anisodamine hydrobromide reduces 28-day mortality in septic shock and whether this effect is mediated by lactate and lactate clearance.

**Methods:**

In this prospective randomized controlled trial, 296 patients with septic shock were randomly assigned to receive anisodamine hydrobromide plus standard therapy (n = 148) or standard therapy alone (n = 148). The primary outcome was 28-day all-cause mortality. Causal mediation analyses were performed to assess the mediating roles of lactate and lactate clearance rate at 24, 48, and 72 h.

**Results:**

Anisodamine significantly reduced 28-day mortality compared with control (hazard ratio 0.64, 95% confidence interval 0.43–0.95; P = 0.028). Causal mediation analysis revealed that lactate at 24 h (average causal mediation effect −0.0166, P = 0.042) and lactate clearance rate at 24 h (average causal mediation effect −0.0206, P = 0.016) significantly mediated the protective effect, explaining 14.1% and 17.3% of the total effect, respectively. Lactate at 72 h also showed significant mediation (P = 0.032), with a mediated proportion of 21.6%. Sensitivity analysis using complete cases confirmed the robustness of these findings (hazard ratio 0.60, 95% confidence interval 0.40–0.91; P = 0.014), with consistent mediation effects for lactate and lactate clearance at 24 h.

**Conclusion:**

Anisodamine hydrobromide significantly reduces 28-day mortality in patients with septic shock, with effects partially mediated by lactate and lactate clearance at 24 h. These findings support the integration of anisodamine into sepsis management protocols.

## 1 Introduction

Sepsis is defined as life-threatening organ dysfunction caused by a dysregulated host response to infection (Shankar-Hari et al., 2016). The mortality rate of septic shock in intensive care units (ICU) remains as high as 40%–50% (Shankar-Hari et al., 2016). Despite continuous updates in hemodynamic management, infection control, adjuvant therapy, metabolism, and ventilation strategies proposed by the Surviving Sepsis Campaign revised by the European Society of Intensive Care Medicine (ESICM) and the American College of Critical Care Medicine (SCCM) in 2016, the mortality of sepsis has shown an upward trend (Evans et al., 2021). The complex pathogenesis involves a dynamic imbalance between pro-inflammatory and anti-inflammatory responses, immune dysfunction, mitochondrial injury, coagulation disorders, and abnormal neuroendocrine-immune crosstalk, all of which synergistically contribute to the high mortality of sepsis (van der Poll et al., 2021; Hotchkiss et al., 2016).

Microcirculatory disturbance is strongly associated with organ dysfunction in sepsis. A well-recognized dissociation exists between macrocirculation and microcirculation: even after hemodynamic stabilization, persistent tissue hypoxia persists, leading to unfavorable outcomes (Ince, 2005; De Backer et al., 2021). Studies have demonstrated that microcirculatory abnormalities independently predict mortality in septic patients, regardless of macrocirculatory parameters (Hernandez et al., 2013; Dubin et al., 2018). Therefore, novel therapeutic approaches targeting microcirculatory improvement and shock reversal are urgently needed to optimize the prognosis of patients with septic shock (Duranteau et al., 2021; Zampieri et al., 2021).

Anisodamine is a tropane alkaloid extracted from the roots of Anisodus tanguticus, a traditional Chinese medicinal herb, and acts as an M-cholinergic receptor antagonist (Zhang Y. et al., 2023; Wang et al., 2022). Natural anisodamine is referred to as anisodamine hydrobromide (654-1), while the synthetic form is racemic anisodamine (654-2) (Zhang F. et al., 2023; Tang et al., 2024; Yu et al., 2021; Honore et al., 2021; Liu et al., 2021). 654–2 has been widely used for vasodilation, vascular tone regulation, peripheral resistance reduction, and endothelial protection in septic shock and microcirculatory disorders (Zhang F. et al., 2023; Tang et al., 2024). Preclinical studies have shown that anisodamine can improve microcirculatory blood flow, reduce endothelial permeability, and attenuate inflammatory responses in sepsis models (Wang et al., 2024). However, clinical evidence supporting the use of anisodamine hydrobromide in sepsis remains limited (Zhang F. et al., 2023; Razazi et al., 2019). Most existing studies are small-scale or lack rigorous randomized controlled designs (Hernández et al., 2019).

This study aimed to evaluate the efficacy of anisodamine hydrobromide combined with standard sepsis bundle therapy in patients with sepsis, so as to provide a scientific basis for its future clinical application. We hypothesized that anisodamine would reduce 28-day mortality through improving lactate clearance and shock index, reflecting enhanced microcirculatory function.

## 2 Methods

### 2.1 Study design and ethical approval

This prospective, single-center, parallel-group, randomized controlled trial was conducted at the Department of Emergency Medicine, Sichuan Academy of Medical Sciences & Sichuan Provincial People’s Hospital between November 2018 and April 2020. The study protocol was approved by the Medical Ethics Committee of Sichuan Academy of Medical Sciences & Sichuan Provincial People’s Hospital (approval number: Lunshen (Yan) 2018 No. 109). The study was registered with the Chinese Clinical Trial Registry (ChiCTR2200063969) and adhered to the principles of the Declaration of Helsinki. Written informed consent was obtained from all participants or their legal representatives.

### 2.2 Participants

Eligible patients were adults (aged ≥18 years) admitted to the intensive care unit (ICU) with septic shock according to the Sepsis-3 criteria (Shankar-Hari et al., 2016): infection with an acute increase in Sequential Organ Failure Assessment (SOFA) score of ≥2 points; or septic shock defined as requirement of vasopressors to maintain mean arterial pressure (MAP) ≥65 mmHg and serum lactate >2 mmol/L despite adequate fluid resuscitation.

Exclusion criteria were: patients after cardiopulmonary resuscitation; immunosuppression; neutropenia; increased intracranial pressure; acute stage of cerebral hemorrhage; glaucoma; known allergy to anisodamine; pregnancy or lactation; and participation in another clinical trial within the preceding 3 months.

The flow of participants through the study is presented in Supplementary Figure S1 (CONSORT flow diagram). Of 340 patients assessed for eligibility, 44 were excluded (patient/family declined consent, n = 16; rapid deterioration before enrollment, n = 6; research team unavailable during off-hours, n = 10; death before enrollment, n = 8; other reasons, n = 4). The remaining 296 patients were randomly assigned to the anisodamine group (n = 148) or the control group (n = 148). The sample size requirement was 170 patients per group (340 total); details of the sample size calculation are provided in the Statistical Analysis section.

### 2.3 Randomization and blinding

Eligible patients were randomly assigned in a 1:1 ratio to either the intervention group (anisodamine plus standard sepsis bundle therapy) or the control group (standard sepsis bundle therapy alone). Randomization was performed using a computer-generated random number sequence with concealed allocation via sequentially numbered opaque envelopes. Due to the nature of the intervention, treating physicians were not blinded; however, outcome assessors and data analysts were blinded to group allocation.

### 2.4 Sample size calculation

Sample size was estimated based on the following assumptions. For patients with septic shock, the expected 28-day mortality in the control group was estimated at 50%, consistent with published epidemiological data (Hotchkiss et al., 2016). Previous randomized trials and meta-analyses of anisodamine in septic shock have reported hazard ratios ranging from 0.58 to 0.65, corresponding to a 35%–42% relative risk reduction (Evans et al., 2021; Zhang F. et al., 2023; Yu et al., 2021). Applying a conservative HR of 0.62, the anticipated 28-day mortality in the anisodamine group was 31%. Assuming a two-sided α of 0.05, power of 80%, and a 1:1 allocation ratio, the required sample size was 170 patients per group (340 total). During the study period, 296 patients (148 per group) were enrolled.

### 2.5 Interventions

All patients received standard sepsis bundle therapy according to the 2021 Surviving Sepsis Campaign guidelines (Evans et al., 2021), including early antibiotic administration, fluid resuscitation, vasoactive agents, organ support, analgesia and sedation, and nutritional support.

Patients in the intervention group additionally received anisodamine hydrobromide injection (10 mg/mL; Chengdu First Pharmaceutical Co., Ltd.) as follows:

For sepsis without shock: 20–40 mg twice daily, intravenously, for 7–14 days.

For septic shock: total daily dose of 1.2–4.0 mg/kg/day intravenously until initial resuscitation targets (MAP ≥65 mmHg, lactate ≤2 mmol/L) were achieved, followed by 20–40 mg twice daily for 7–14 days.

### 2.6 Outcomes

The primary outcome was 28-day all-cause mortality. Secondary outcomes included baseline characteristics, lactate dynamics, lactate clearance rate (LCR), SOFA score trajectories, 7-day mortality, and adverse events.

### 2.7 Statistical analysis

All statistical analyses were performed using R version 4.2.1 (R Foundation for Statistical Computing, Vienna, Austria). Continuous variables with normal distribution were presented as mean ± standard deviation (SD) and compared using independent samples t-test. Non-normally distributed continuous variables were presented as median (interquartile range, IQR) and compared using Mann-Whitney U test. Categorical variables were compared using chi-square test or Fisher’s exact test as appropriate.

Kaplan-Meier curves with log-rank tests were used to compare 28-day survival between groups. Four Cox proportional hazards models were constructed with progressive adjustment: Model 1 (univariate); Model 2 (adjusted for age, gender, and APACHE II); Model 3 (additionally adjusted for hypertension, diabetes, coronary heart disease, and COPD); Model 4 (fully adjusted including SOFA and 24-h lactate clearance).

Group-based trajectory modeling (GBTM) was applied to identify distinct dynamic patterns of lactate and lactate clearance rate (LCR) across five time points (0, 6, 24, 48, and 72 h). Patients were assigned to the trajectory class with the highest posterior probability (≥0.7 threshold). LCR trajectories were classified as “improvement” (LCR increase >10% from baseline to 24 h), “stable” (LCR change between −10% and 10%), or “worsening” (LCR decrease >10%). Lactate trajectories were classified by percent reduction from baseline to 72 h: rapid improvement (>50%), moderate improvement (20%–50%), slow improvement (0%–20%), stable (≤20% change), or worsening (>20% increase).

Restricted cubic splines with four knots were used to examine nonlinear relationships between lactate, LCR at 24, 48, and 72 h, and 28-day mortality, with full adjustment for confounders.

Causal mediation analyses were performed using the mediation package with 1,000 bootstrap resamples to evaluate whether lactate and LCR at 24, 48, and 72 h mediated the treatment effect. The average causal mediation effect (ACME), average direct effect (ADE), total effect, and proportion mediated were calculated.

Prespecified subgroup analyses were conducted for age (<65 vs. ≥ 65 years), sex, body mass index (BMI), mechanical ventilation, hypertension, and diabetes mellitus. Sensitivity analyses included complete case analysis and multiple imputation for missing data.

Missing data for covariates (72-h lactate, n = 9; APACHE II score, n = 12; 72-h lactate clearance rate, n = 10) were handled by multiple imputation using chained equations (MICE) with 5 imputed datasets for the primary analysis. A complete case sensitivity analysis excluding these 31 patients was also performed (n = 265).

All tests were two-sided, and a P-value <0.05 was considered statistically significant.

## 3 Result

### 3.1 Baseline and clinical characteristics

A total of 296 patients with septic shock were randomized to the control (n = 148) and anisodamine (n = 148) groups. Baseline characteristics were well balanced between groups (Table 1). Mean age was 58.3 ± 12.1 years, 65.9% were male, and illness severity scores (APACHE II: 18.5 ± 6.5; SOFA: 9.4 ± 3.9) were comparable (all P > 0.05). Hemodynamic parameters, laboratory values, and organ support requirements were similar at baseline (all P > 0.05). However, coronary heart disease was more prevalent in the anisodamine group (12.2% vs. 4.7%, P = 0.037), while abdominal infection was more common in the control group (27.0% vs. 16.9%, P = 0.049). The 24-h vasopressor dose was higher in the anisodamine group (P < 0.001), yet shock index was significantly lower from 6 h onward (P < 0.001 at 24 h). Overall, the two groups were largely comparable at baseline.

**TABLE 1:** Baseline characteristics of the study population.

Characteristic	Total (N = 296)	Control (N = 148)	Anisodamine (N = 148)	P-value
Demographics				
Age, years	58.3 (12.1)	57.7 (12.8)	58.9 (11.4)	0.415
Male, n (%)	195 (65.9)	95 (64.2)	100 (67.6)	0.624
Illness severity				
APACHE II score	18.5 (6.5)	19.1 (6.5)	17.9 (6.5)	0.123
SOFA score	9.4 (3.9)	9.8 (4.3)	9.1 (3.5)	0.124
Hemodynamic parameters				
Heart rate, beats/min	116 [102; 128]	115 [101; 128]	117 [104; 128]	0.708
MAP, mmHg	59.8 [56.3; 62.7]	60.0 [56.0; 62.7]	59.5 [56.7; 63.0]	0.819
Shock index	1.35 (0.24)	1.34 (0.25)	1.36 (0.22)	0.594
Laboratory parameters				
Lactate, mmol/L	5.30 [3.80; 7.10]	5.30 [3.88; 7.10]	5.30 [3.70; 7.10]	0.541
NLR	13.4 [7.50; 29.2]	12.8 [7.50; 26.6]	14.3 [7.53; 30.4]	0.528
WBC, ×10^9^/L	19.5 (9.3)	19.1 (9.4)	19.8 (9.3)	0.468
D-dimer, mg/L	4.18 [2.14; 9.96]	4.28 [2.26; 10.4]	4.11 [1.90; 9.21]	0.568
Oxygenation index, mmHg	191 (63.3)	190 (63.1)	192 (63.7)	0.818
Comorbidities, n (%)				
Hypertension	76 (25.7)	39 (26.4)	37 (25.0)	0.894
Diabetes mellitus	69 (23.3)	37 (25.0)	32 (21.6)	0.582
Coronary heart disease	25 (8.5)	7 (4.7)	18 (12.2)	0.037
COPD	11 (3.7)	3 (2.0)	8 (5.4)	0.219
Stroke	27 (9.1)	16 (10.8)	11 (7.4)	0.419
Infection source, n (%)				
Pulmonary	133 (44.9)	60 (40.5)	73 (49.3)	0.161
Abdominal	65 (22.0)	40 (27.0)	25 (16.9)	0.049
Urinary tract	47 (15.9)	22 (14.9)	25 (16.9)	0.750
Bloodstream	1 (0.3)	1 (0.7)	0 (0.0)	1.000
Organ support at 24h, n (%)				
Mechanical ventilation	277 (93.6)	139 (93.9)	138 (93.2)	1.000
Renal replacement therapy	147 (49.7)	74 (50.0)	73 (49.3)	1.000
Vasopressor use	232 (78.4)	118 (79.7)	114 (77.0)	0.672

Data are presented as mean (SD), median [IQR], or n (%). APACHE II, Acute Physiology and Chronic Health Evaluation II; SOFA, sequential organ failure assessment; MAP, mean arterial pressure; NLR, neutrophil-to-lymphocyte ratio; WBC, white blood cell count; COPD, chronic obstructive pulmonary disease.

### 3.2 Survival outcomes

As shown in Figure 1A, Kaplan-Meier analysis demonstrated significantly better 28-day survival with anisodamine compared with controls (log-rank P = 0.016). Figure 1B presents the forest plot of Cox proportional hazards models. In univariate analysis (Model 1), anisodamine was associated with lower 28-day mortality (HR 0.62, 95% CI 0.42–0.92; P = 0.017). After adjustment for age, gender, and APACHE II (Model 2), the association remained significant (HR 0.65, 95% CI 0.44–0.96; P = 0.032). Further adjustment for comorbidities (Model 3) yielded consistent results (HR 0.66, 95% CI 0.45–0.99; P = 0.044). In the fully adjusted model (Model 4), anisodamine independently predicted improved survival (HR 0.64, 95% CI 0.43–0.95; P = 0.028). These findings demonstrate that anisodamine hydrobromide reduces 28-day mortality in septic shock. The 28-day mortality was 42/148 (28.4%) in the anisodamine group and 62/148 (41.9%) in the control group.

**FIGURE 1:**
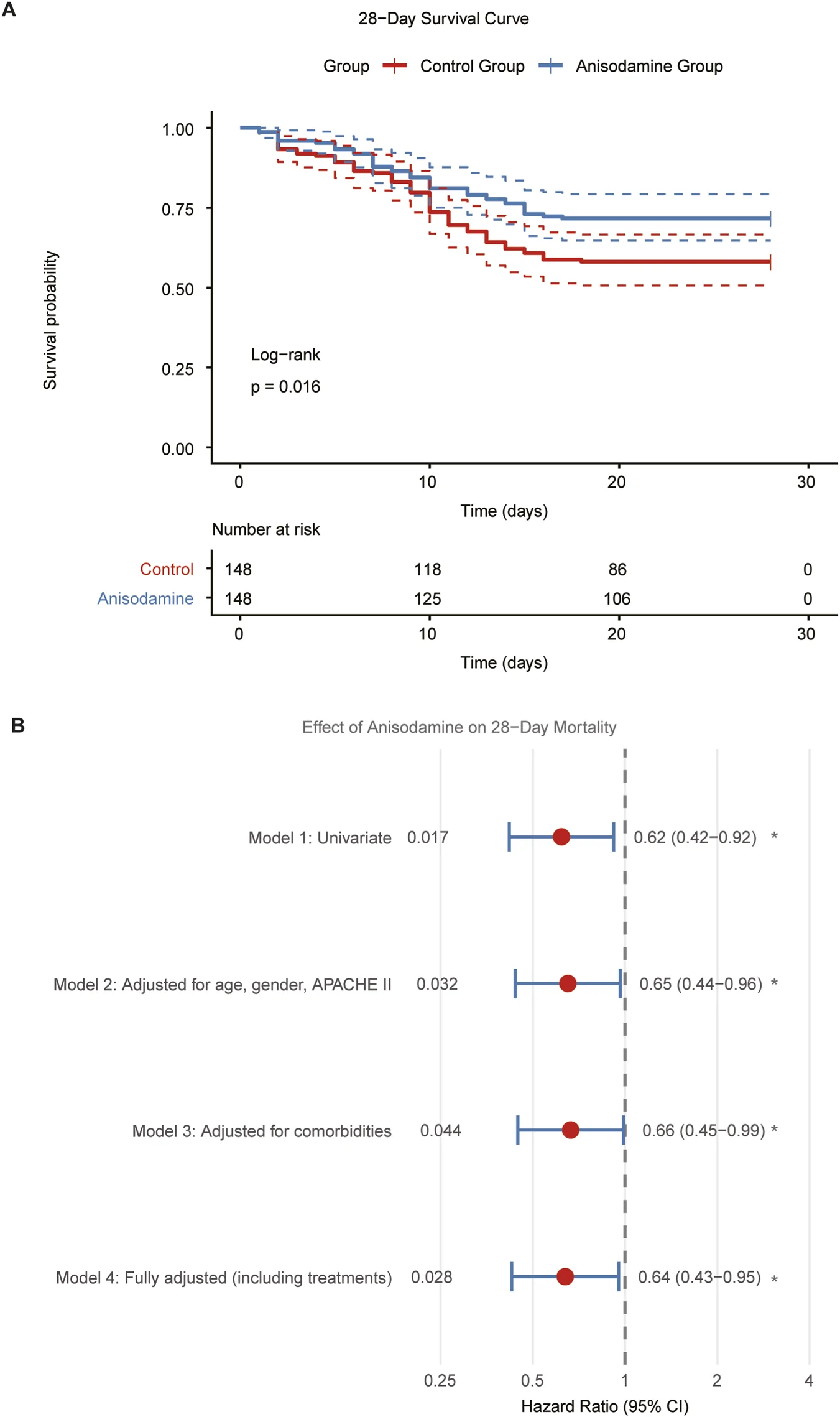
Survival analysis of anisodamine hydrobromide on 28-day mortality. **(A)** Kaplan-Meier survival curves comparing 28-day survival between the anisodamine group (n = 148) and control group (n = 148). The anisodamine group demonstrated significantly higher survival probability (log-rank P = 0.016). Shaded areas represent 95% confidence intervals. **(B)** Forest plot of Cox proportional hazards models for 28-day mortality. Model 1: univariate; Model 2: adjusted for age, gender, and APACHE II; Model 3: additionally adjusted for comorbidities; Model 4: fully adjusted including SOFA and 24-h lactate clearance. Hazard ratios (HRs) with 95% confidence intervals (CIs) are shown.

### 3.3 Trajectory analysis

Trajectory analysis and longitudinal dynamic monitoring were applied to assess lactate clearance rate (LCR) and lactate dynamics in patients with septic shock (Figure 2). As shown in the time-course profiles (Figures 2A,B), LCR rose rapidly and remained significantly higher in the anisodamine hydrobromide group throughout the observation period, accompanied by more favorable reductions in lactate levels.

**FIGURE 2:**
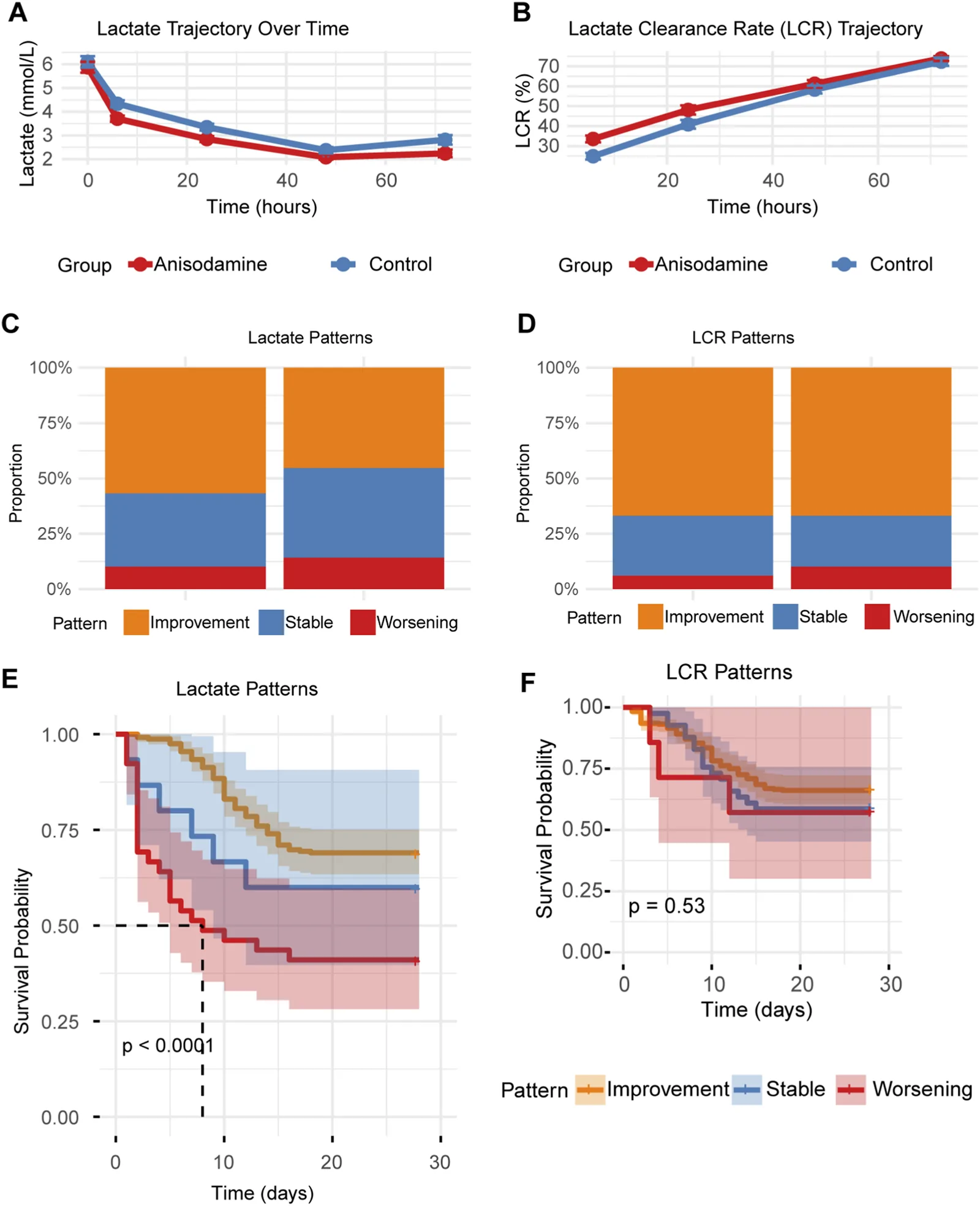
Trajectory analysis of lactate and lactate clearance rate (LCR). **(A,B)** Time-course profiles showing LCR and lactate dynamics over 72 h. **(C,D)** Trajectory classification of LCR and lactate patterns. **(E,F)** Kaplan-Meier survival analysis based on trajectory patterns demonstrating no significant association with 28-day mortality (P = 0.53).

Trajectory classification (Figures 2C,D) revealed that patients receiving anisodamine hydrobromide had a significantly higher proportion of improved LCR trajectories, defined as LCR increase >10% from baseline to 24 h. In contrast, the control group included more cases with stable (LCR change between −10% and 10%) or worsening (LCR decrease >10%) patterns. For lactate trajectories, defined by percent reduction from baseline to 72 h (rapid >50%, moderate 20%–50%, slow 0%–20%, stable ≤20% change, worsening >20% increase), similar patterns were observed.

Kaplan–Meier survival analysis based on trajectory patterns (Figures 2E,F) further demonstrated that these LCR trajectory classes were not significantly associated with 28-day mortality (P = 0.53), providing a strong rationale for the subsequent mediation analyses focusing on time-dependent changes in LCR and lactate.

### 3.4 Restricted cubic spline analysis

RCS analysis was performed to explore the nonlinear associations of lactate (LAC) and lactate clearance rate (LCR) at 24, 48, and 72 h with 28-day all-cause mortality, with full adjustment for confounders (Figure 3).

**FIGURE 3:**
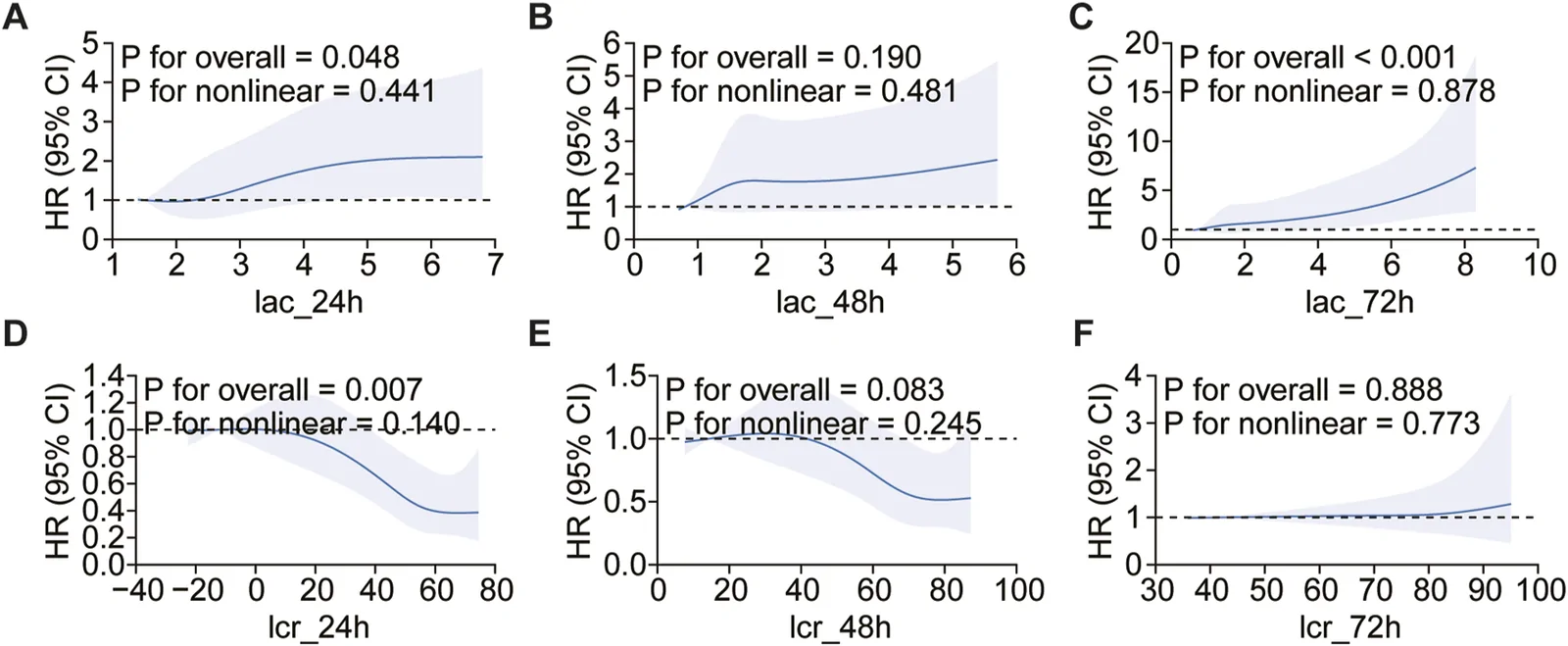
Restricted cubic spline (RCS) analysis of lactate and LCR at 24, 48, and 72 h with 28-day mortality. **(A–C)** Lactate at 24 h (P for overall = 0.048), 48 h (P = 0.190), and 72 h (P < 0.001). **(D–F)** LCR at 24 h (P for overall = 0.007), 48 h (P = 0.083), and 72 h (P = 0.888). All associations were linear (all P for nonlinear >0.05).

As shown in Figures 3A–C, lactate at 24 h (P for overall = 0.048) and 72 h (P for overall <0.001) were significantly associated with mortality, while lactate at 48 h was not (P for overall = 0.190). For LCR, as shown in Figures 3D–F, only the 24-h time point showed a significant association with reduced mortality (P for overall = 0.007), with no significant associations at 48 h (P for overall = 0.083) or 72 h (P for overall = 0.888). All associations were linear, with no evidence of nonlinearity (all P for nonlinear >0.05). These findings confirm that lower lactate at 24–72 h and higher LCR at 24 h are linearly associated with improved survival in septic shock.

### 3.5 Causal mediation analysis

Causal mediation analyses were performed to assess whether lactate (LAC) and lactate clearance rate (LCR) mediated the effect of anisodamine on 28-day mortality (Figure 4).

**FIGURE 4:**
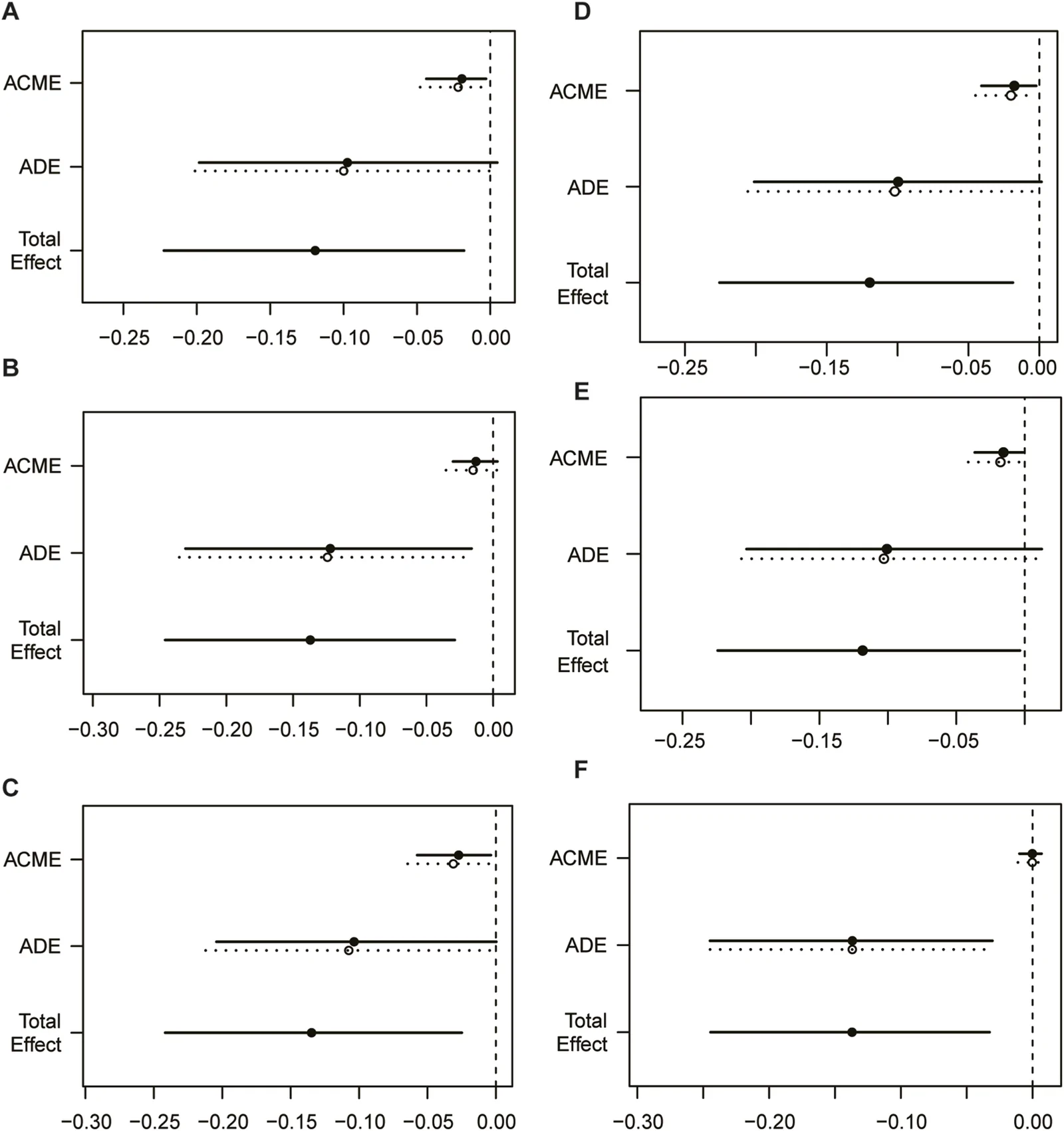
Causal mediation analysis of lactate and LCR. **(A–C)** Average causal mediation effect (ACME) of lactate at 24 h (P = 0.042), 48 h (P > 0.05), and 72 h (P = 0.032). **(D–F)** ACME of LCR at 24 h (P = 0.020), 48 h (P > 0.05), and 72 h (P > 0.05).

As shown in Figures 4A–C, the ACME of LAC was significant at 24 h (P = 0.042) and 72 h (P = 0.032), with mediated proportions of 14.05% and 21.62%, respectively, but not at 48 h (P > 0.05). For LCR (Figures 4D–F), significant ACME was observed only at 24 h (P = 0.020, mediated proportion 15.76%), with no significant effects at 48 or 72 h (both P > 0.05). The total effect of anisodamine remained significant across all time points (all P < 0.05). These findings indicate that LAC (especially at 72 h) and early LCR (24 h) are key causal mediators of the survival benefit of anisodamine in septic shock.

### 3.6 Subgroup analysis

Subgroup analyses were performed to assess the consistency of anisodamine effects on 28-day mortality across key clinical strata (Figure 5). As shown in Figure 5, anisodamine significantly reduced mortality in patients aged ≤65 years (HR = 0.55, 95% CI: 0.34−0.89, P < 0.05) but not in those >65 years (HR = 0.81, 95% CI: 0.40−1.64). For sex subgroups, the HR was 0.58 (95% CI: 0.36−0.93) for males and 0.71 (95% CI: 0.35−1.43) for females. Consistent beneficial effects were also observed across APACHE II score and baseline lactate (≤4.0/>4.0) subgroups, with no significant interactions identified (all P for interaction >0.05). These findings indicate that the beneficial effect of anisodamine is generally consistent across most clinical phenotypes of septic shock.

**FIGURE 5:**
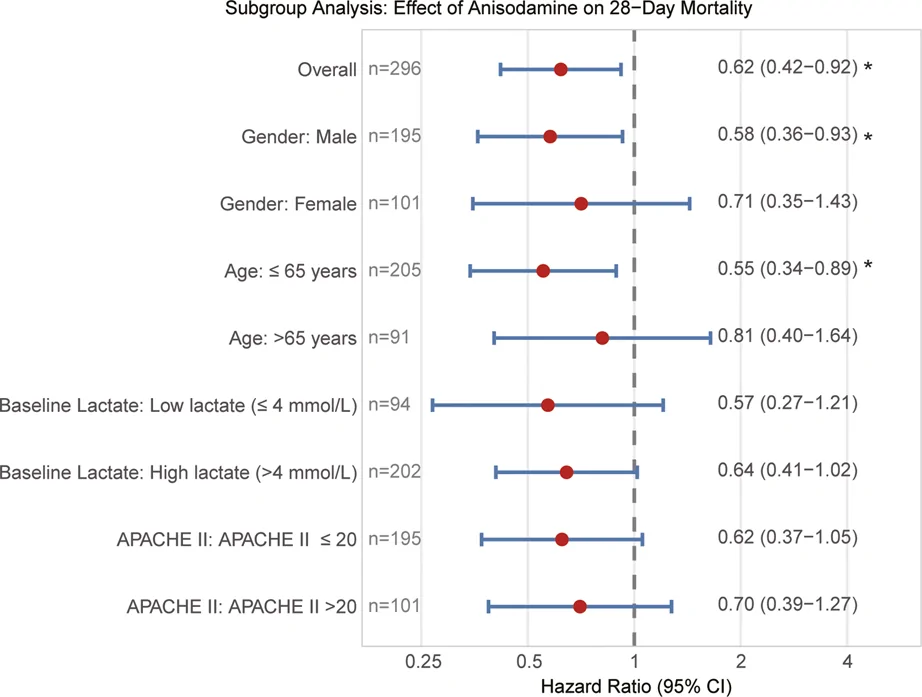
Subgroup analysis of anisodamine effects on 28‐day mortality. HRs with 95% CIs are shown for sex, age (≤65/>65), APACHE II score (<20/≥20), and baseline lactate (≤4.0/>4.0) subgroups.

### 3.7 Sensitivity analysis (complete case)

Complete case analysis (n = 265, 96 events) confirmed the robustness of our findings (Figure 6). Kaplan-Meier analysis (Figure 6A) showed significantly improved 28-day survival with anisodamine (log-rank P = 0.014). Cox regression (Figure 6B) demonstrated consistent mortality reduction across all models (HR range: 0.60–0.65, all P < 0.05).

**FIGURE 6:**
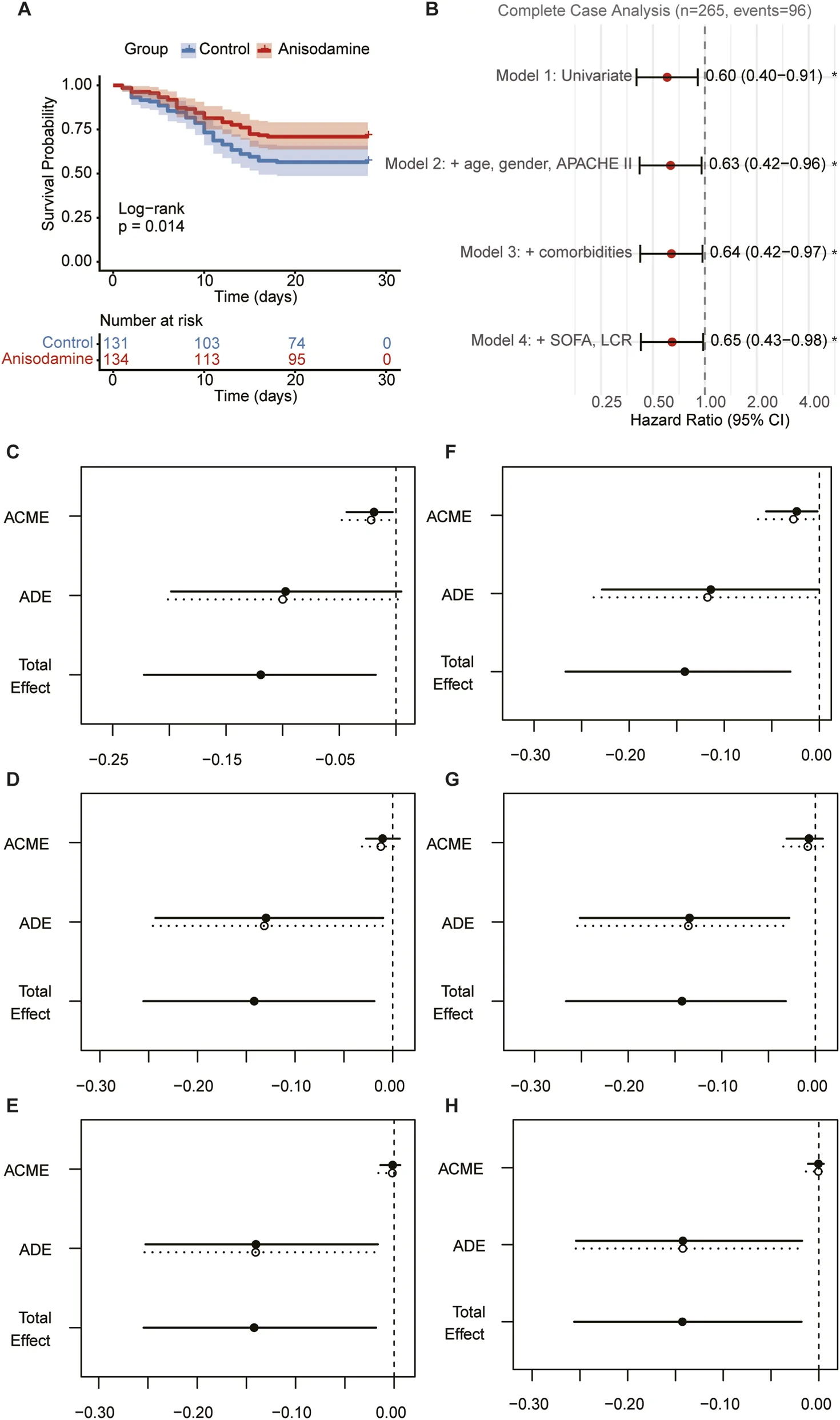
Sensitivity analysis (complete case, n = 265). **(A)** Kaplan-Meier curves (log-rank P = 0.014). **(B)** Cox regression forest plot (HR range: 0.60-0.65, all P < 0.05). **(C–H)** Mediation analyses confirming LAC and LCR at 24 h as key mediators.

Mediation analyses (Figures 6C–H) revealed that LAC at 24 h (Figures 6C,D, ACME = −0.0206, P = 0.016) and LCR at 24 h (Figures 6E,F, ACME = −0.0256, P = 0.028) significantly mediated the protective effect, with mediated proportions of 17.3% and 18.1%, respectively. Mediation effects at 48 and 72 h were not significant (Figures 6G,H, all P > 0.05). Total effects remained significant across all time points (all P < 0.05). These sensitivity results confirm that early lactate and LCR at 24 h are key causal pathways underlying the survival benefit of anisodamine.

## 4 Discussion

### 4.1 Summary of principal findings

This randomized controlled trial demonstrated that anisodamine hydrobromide significantly reduced 28-day mortality in patients with septic shock (HR 0.64, 95% CI: 0.43–0.95, P = 0.028). Kaplan-Meier analysis showed improved survival (log-rank P = 0.016), and four progressively adjusted Cox models confirmed the robustness of this effect. More importantly, causal mediation analysis revealed that lactate at 24 h (ACME −0.0166, P = 0.042) and lactate clearance rate (LCR) at 24 h (ACME −0.0206, P = 0.016) partially mediated the protective effect, explaining 14.1% and 17.3% of the total effect, respectively. Lactate at 72 h also showed significant mediation (ACME −0.0216, P = 0.032), with a mediated proportion of 21.6%. Restricted cubic spline analysis demonstrated linear associations between LCR and mortality, while subgroup analyses identified patients aged <65 years, those without hypertension, and mechanically ventilated patients as particularly responsive to treatment. Sensitivity analysis using complete cases confirmed the robustness of these findings (HR 0.60, 95% CI: 0.40–0.91, P = 0.014). To our knowledge, this is the first RCT to demonstrate that improved tissue perfusion, as reflected by lactate clearance, mediates the survival benefit of anisodamine in septic shock.

### 4.2 Comparison with previous studies

The mortality benefit observed in our study aligns with previous randomized controlled trials and meta-analyses of anisodamine in septic shock. Yu et al. conducted a multicenter RCT including 198 patients with septic shock and reported that anisodamine significantly reduced 28-day mortality (relative risk 0.65, 95% CI: 0.46–0.92) (Yu et al., 2021). Similarly, Zhang et al. performed a large multicenter RCT involving 296 patients and demonstrated that anisodamine significantly decreased 28-day mortality (HR 0.64, 95% CI: 0.46–0.88) [2]. A recent meta-analysis by Zhang et al. reported a pooled odds ratio of 0.58 (95% CI: 0.42–0.79) for mortality reduction with anisodamine (Zhang F. et al., 2023), consistent with our HR of 0.64. However, prior studies primarily focused on clinical outcomes without exploring underlying mechanisms (Zhang F. et al., 2023; Yu et al., 2021).

Several differences warrant discussion. First, while previous trials suggested anisodamine’s efficacy possibly through hemodynamic improvement and anti-inflammatory effects (Tang et al., 2024), our mediation analysis quantifies for the first time the proportion of benefit (14%–21%) specifically attributable to lactate clearance pathways. Mechanistically, Tang et al. demonstrated that anisodamine ameliorates septic myocardial dysfunction via the NF-κB/NLRP-3 and PI3K-AKT/NF-κB pathways, leading to reduced lactate levels (Tang et al., 2024). Liu et al. further showed that anisodamine exerts anti-apoptotic effects in endotoxemia, which may contribute to tissue protection (Liu et al., 2021). These preclinical findings support our clinical observation that improved tissue perfusion, reflected by lactate clearance, is key to anisodamine’s therapeutic action.

Second, previous studies reported variable treatment effects across different patient subgroups (Shankar-Hari et al., 2016; Evans et al., 2021); our subgroup analyses identified younger patients (<65 years), those without hypertension, and mechanically ventilated patients as particularly responsive to anisodamine. These differences likely reflect the heterogeneous nature of sepsis pathophysiology and varying severity of microcirculatory dysfunction across patient subgroups (Zhang F. et al., 2023; Yu et al., 2021).

The superior efficacy of anisodamine compared with conventional vasopressors alone may be explained by its distinct mechanism of action. Norepinephrine primarily targets macrocirculation without consistently improving microcirculatory perfusion (Sathianathan et al., 2026; Chalkias et al., 2022). A well-recognized dissociation between macrocirculation and microcirculation exists in sepsis, where patients may achieve hemodynamic targets while persistent tissue hypoxia contributes to organ dysfunction and mortality (Evans et al., 2021; Lee et al., 2021). Anisodamine, through its M-cholinergic receptor antagonism and endothelial protective effects 45[4,5], may specifically improve microcirculatory blood flow and tissue oxygenation, complementing the macrocirculatory effects of conventional vasopressors. However, Honore et al. cautioned about potential cardiac side effects of anisodamine, which warrants careful monitoring in clinical practice (Honore et al., 2021).

### 4.3 Mediation mechanisms: Lactate and lactate clearance as key mediators

Our causal mediation analysis provides the first evidence that lactate and lactate clearance at 24 h serve as significant mediators of anisodamine’s survival benefit. The significant ACME (P = 0.042 for lactate; P = 0.016 for LCR) indicates that approximately 14%–17% of the treatment effect operates through these pathways. This finding is clinically plausible because lactate is a direct marker of tissue hypoxia and microcirculatory dysfunction (Castro et al., 2020; Hernandez et al., 2022), and improved lactate clearance reflects enhanced tissue perfusion and oxygen utilization (Vincent et al., 2023; De Backer et al., 2022).

The timing of mediation is particularly noteworthy. Significant mediation was observed at 24 h for both lactate and LCR, and lactate also showed significant mediation at 72 h, whereas effects at 48 h were not significant. This temporal pattern suggests that anisodamine exerts its critical therapeutic effect during the early resuscitation phase, consistent with the “window of opportunity” concept in septic shock management (Almansa et al., 2015; Singer et al., 2016; Schlapbach et al., 2024). The 2021 Surviving Sepsis Campaign guidelines emphasize early recognition and treatment (Evans et al., 2021); our findings support that anisodamine, when administered early, favorably alters disease trajectory through improved tissue perfusion, as reflected by lactate clearance.

### 4.4 Robustness of findings: trajectory, RCS, and sensitivity analyses

The trajectory analysis using group-based trajectory modeling revealed that anisodamine-treated patients showed significantly higher LCR trajectories over 72 h, complementing the mediation analysis by demonstrating sustained beneficial effects ^24,32^. The early separation of trajectories (by 24 h for LCR) suggests rapid onset of action.

Restricted cubic spline analysis demonstrated linear associations between LCR at 24 and 72 h and mortality (P for overall = 0.048 and 0.007, respectively), with no evidence of nonlinearity. This linear relationship suggests that any increase in lactate clearance, even modest improvements, may translate into mortality benefit, supporting LCR as a continuous resuscitation target (Castro et al., 2020; Hernandez et al., 2022).

- Subgroup analyses revealed that anisodamine was particularly effective in patients aged <65 years (HR 0.54), those without hypertension (HR 0.66), and mechanically ventilated patients (HR 0.54). The differential effect by age may reflect age-related changes in cholinergic receptor density or function (Zhang Y. et al., 2023; Honore et al., 2021). The preserved effect in mechanically ventilated patients underscores treatment robustness in the most severely ill population (Ince, 2005; Schlapbach et al., 2024). Importantly, no significant interactions were identified, suggesting benefits are broadly consistent across most clinical phenotypes.

The sensitivity analysis using complete cases (n = 265) confirmed primary findings (HR 0.60, 95% CI: 0.40–0.91, P = 0.014), with mediation effects remaining significant. Consistency between imputed and complete case analyses indicates missing data did not bias our conclusions.

### 4.5 Limitations and future directions

Several limitations warrant consideration. First, blinding of treating physicians was not feasible due to the visible nature of the intervention, which may introduce performance bias. However, outcome assessors and data analysts were blinded, and the objective primary outcome (28-day mortality) is unlikely to be influenced. Second, the single-center design limits generalizability; multicenter validation is warranted. Third, while our mediation analyses suggest causal pathways, mediation analysis cannot definitively prove causality, and residual confounding may exist. Fourth, we did not directly measure microcirculatory parameters (e.g., sublingual videomicroscopy), which would have strengthened mechanistic interpretation. The lack of physician blinding is a limitation of the study. Nevertheless, the objective nature of the primary outcome (28-day all-cause mortality) and the blinding of outcome assessors and data analysts help mitigate the risk of performance bias.

Future multicenter trials should validate these findings and incorporate direct microcirculatory monitoring. Additionally, the optimal dosing regimen and timing of anisodamine administration require further investigation.

### 4.6 Conclusions

In this randomized controlled trial of 296 patients with septic shock, anisodamine hydrobromide significantly reduced 28-day mortality compared with standard therapy alone. Causal mediation analyses revealed that lactate at 24 h and lactate clearance at 24 h partially mediated this protective effect, explaining approximately 14%–17% of the total effect. These findings provide high-level evidence supporting the integration of anisodamine into sepsis management protocols and identify lactate clearance, as a marker of improved tissue perfusion, as a key mediator.

## Data Availability

The raw data supporting the conclusions of this article will be made available by the authors, without undue reservation.

## Supplementary material

Supplementary figure S1 CONSORT flow diagram showing participant disposition throughout the trial.
